# Effects of landscape complexity and stand factors on arthropod communities in poplar forests

**DOI:** 10.1002/ece3.5285

**Published:** 2019-06-11

**Authors:** Binli Wang, Chengming Tian, Jianlong Sun

**Affiliations:** ^1^ The Key Laboratory for Silviculture and Conservation of Ministry of Education, College of Forestry Beijing Forestry University Beijing China

**Keywords:** arthropod communities, biological control, defoliators, landscape complexity, natural enemies, poplar forest, stand factors

## Abstract

The arthropod communities are influenced by both local conditions and features of the surrounding landscape. Landscape complexity and stand factors may both influence arthropod communities in poplar forests, but the multiscale effects of these factors on poplar defoliators and natural enemies are still poorly understood. We collected poplar arthropods at 30 sampling sites within five forest landscapes in Xinjiang, China, and assessed whether landscape complexity and stand factors influence species abundance and diversity of poplar arthropods. Landscape complexity was quantified by several independent metrics of landscape composition, configuration, and connectivity at three spatial scales. We also determined the most powerful explanatory variables and the scale effect of each arthropod. Results found that landscape complexity and stand factors had different effects on different poplar arthropod communities. Landscape complexity promoted natural enemies at different spatial scales, but it inhibited the population of poplar defoliators at the scale of 200 m. Specifically, the abundance and diversity of all defoliators decreased with increasing proportion of nonhost plants. Landscape diversity only had a negative effect on defoliator abundance. The shape complexity of habitat patches increased the abundance of carabid beetles but reduced the abundance of green leafhoppers and migratory locusts. The abundance and diversity of predators increased with increasing structural connectivity of forest landscape. Additionally, both the abundance and diversity of all defoliators were positively correlated with the average height of herbaceous plants. Diversity of all defoliators increased with increasing size of host trees. The distance from sampling site to the nearest village positively influenced the abundance and diversity of all predators. Arthropod abundance and diversity in poplar forests were driven by stand factors and landscape complexity. Therefore, maintaining complex shape and structural connectivity of habitat patches and keeping poplar stands away from the village are crucial for management of forest landscape to enhance natural enemies. And in order to reduce the abundance of defoliators in poplar forest, the diversity of surrounding habitat types should be promoted within 200 m radii.

## 1 INTRODUCTION

An overreliance on chemical control not only leads to a resistance risk of insect pests and to a reduction in natural enemies but also poses numerous threats to the ecological environment security (Steinmann et al., 2010). As an important ecosystem service, biological control may offer a sustainable solution to pest problems and conserving it requires knowledge of the structure of arthropod community and of the effects of different environmental factors acting at the local and landscape scales. (Chaplin‐Kramer, O'Rourke, Blitzer, & Kremen, 2011; Losey & Vaughan, 2006; Martin, Reineking, Seo, & Steffan‐Dewenter, 2013; Schneider, Krauss, Riedinger, Holzschuh, & Steffan‐Dewenter, 2015). It has been demonstrated that the abundance and diversity of arthropods are influenced by several factors, such as local vegetation management, stand characteristics, and landscape complexity of the surrounding environment (Balzan, Bocci, & Moonen, 2015; Li, Zhou, & Wu, 1998; Tscharntke et al., 2007; Wermelinger et al., 2017).

Among these factors, plenty of studies suggest that increasing landscape complexity generally enhance natural enemy abundance and/or diversity; however, pest responses to landscape complexity are much less determinate (Chaplin‐Kramer et al., 2011; Drapela, Moser, Zaller, & Frank, 2008; Rusch et al., 2013; Schirmel et al., 2018; Schmidt, Thies, Nentwig, & Tscharntke, 2008). The effects of landscape complexity on arthropods have been explored across a range of study regions and ecological systems and the definition of landscape complexity varies widely from study to study, but landscape structure may influence arthropod populations by a number of ways (Fahrig et al., 2011), this is often referred to as landscape complexity (Macfadyen, Kramer, Parry, & Schellhorn, 2015). Three components determine landscape complexity: (a) landscape composition (the variety of different habitat types); (b) landscape configuration (the number, size, and shape of landscape elements; Concepción, Díaz, & Baquero, 2008; Duelli, 1997); and (c) landscape connectivity (the connectivity of habitat patches; Macfadyen et al., 2015). Three components of landscape complexity all have different ecological implications for arthropod communities (Macfadyen et al., 2015; Pasher et al., 2013). A growing body of evidence shows that the complexity in composition (the diversity of habitat types) or configuration (the spatial arrangement of habitat patches) of a landscape influence pests, natural enemies, and other arthropod communities of an ecosystem (Balzan et al., 2015; Dominik et al., 2018; Haenke et al., 2014; Rösch, Tscharntke, Scherber, Batáry, & Osborne, 2013), and they especially can enhance natural enemy diversity or abundance (Chaplin‐Kramer et al., 2011; Gallé et al., 2018; Schirmel et al., 2018). The connectivity of a landscape also gains importance to natural enemies because it allows them more freedom of movement and enhances their ability to migrate in search of food and safety (Baggio, Salau, Janssen, Schoon, & Bodin, 2011; Coppolillo, Gomez, Maisels, & Wallace, 2004; Diekötter, Billeter, & Crist, 2008).

The Irtysh River, the chief tributary of the Ob River, arises from the glaciers on the southwestern slopes of the Altai Mountains in the Uygur Autonomous Region of Xinjiang in far northwestern China. Natural poplar forests widely distributed on both banks of this river are mainly *Populus alba* L., *Populus nigra* L., and* Populus laurifolia* Ledeb. These trees are one of the most productive components of riparian ecosystem, shaping the ecology of such a fragile environment (Cartisano et al., 2013). Owing to their large sizes and long lifespans, many *Populus* species are constantly attacked by a wide variety of insect herbivores (e.g., defoliators, shoot feeders, and stem borers; Philippe & Bohlmann, 2007), but only a few species are responsible for heavy damage in natural poplar forests. Even though defoliators form the largest proportion of insect herbivores, most of them seldom cause mortality of trees (Kosola, Dickmann, Paul, & Parry, 2001). The capacity of poplar trees to cope with huge numbers of potential insect herbivores over the years is mainly driven by the conservation of natural enemies. This may be due to the fact that local herdsmen mowed the aboveground plants regularly every year and even destroyed the poplar seedlings, coupled with the expansion of river closure lead to a significant decline of woodland, making forest landscape increasingly complex. Hence, natural enemies and the level of biological control they provide can be enhanced by complex features of surrounding landscape (Schirmel et al., 2018). Recent findings have provided evidence that generalist natural enemies may be more effective than specialists in pest control (Björkman, Dalin, & Eklund, 2003; Chaplin‐Kramer et al., 2011). Many carabid beetles, lacewings, and all spiders are polyphagous predators and are widely used in biological control programs (Bertrand, Burel, & Baudry, 2016; Gallé et al., 2018). An essential step toward effective biological control is to identify the important natural enemy species, and efficient biological control requires a landscape perspective because the surrounding landscape may provide alternative food sources, overwintering sites, and a refuge to natural enemies (Balzan et al., 2015; Björkman et al., 2003). But most studies focused on the potential benefits of landscape complexity for natural enemies in agrosystems, only a few studies researched the generalist predators of poplar defoliators (Dominik et al., 2018; Schirmel et al., 2018).

Many previous studies reported that forest stand factors can affect arthropods, and furthermore, they influenced the population dynamics of herbivores and natural enemies (Alalouni, Brandl, Auge, & Schädler, 2014; Bach, 1980). At stand level, the canopy density and ground vegetation conditions contribute to the population dynamics of the pine caterpillar (Li et al., 1998). Stand characteristics relevant to increased abundance of cockchafer grubs included dense vegetation cover and moderate canopy openness (Niemczyk, Karwański, & Grzybowska, 2017). Stand factors driving herbivore population dynamics may also affect their natural enemies. Drapela et al. (2008) showed that site characteristics (e.g., stand density and late autumn ground cover) and landscape factors were similarly important for explaining species composition of spiders. To date, however, less is known about the way in which stand factors (such as ground vegetation conditions, size of host trees, and the distance from sampling site to the nearest river and the nearest village) have shaped poplar arthropod communities at broader scales or within the context of landscape complexity. Furthermore, determining which landscape‐scale and stand‐scale factors are significantly related to the abundance or diversity of arthropod communities would provide valuable information to forest managers deciding what management techniques are important to the development of biological control (Hannah et al., 2017).

Therefore, for the first time, we collect poplar arthropods at 30 sampling sites within 5 forest landscapes in Xinjiang, China, and quantify landscape complexity using several landscape metrics of composition, configuration, and connectivity at three spatial scales. Because this is the first study to examine the combined effects of landscape complexity and stand factors on poplar arthropod communities, particularly the natural enemies, we also determine the spatial extent at which these factors best predicts abundance and diversity of each arthropod. Such scale of effect would enable us to work at the most effective spatial scale (Jackson & Fahrig, 2012). Specifically, we hypothesized that (1) the compositional landscape complexity (the diversity of different habitat types) increases the abundance and diversity of natural enemies, (2) the configurational landscape complexity (size and shape of habitat patches) has positive effects on natural enemy communities, (3) the abundance and diversity of natural enemies respond positively to the connectivity of forest landscape; and (4) the abundance and diversity of different poplar arthropods respond differently to several stand factors.

## 2 MATERIALS AND METHODS

### 2.1 Study regions and site selection

**Figure 1:**
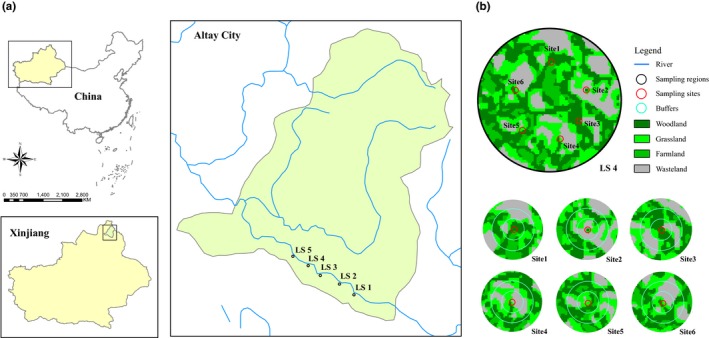
Study area on the southern bank of the Irtysh River in Xinjiang, China. (a) Distribution of the five circular sampling regions of 0.5‐km radius in Jiaosate (LS1), Akeqiapuba (LS2), Yonghong (LS3), Kezierhaying (LS4), and Mahulemuhanarele (LS5). (b) Locations of the six sampling sites of each landscape, and examples of mapping land cover features within 100, 200, and 300 m radii buffers around sampling sites, take LS4, for example

The study was conducted in natural poplar forests across about 40 km long, which distributed on the southern bank of the Irtysh River in Xinjiang, China. Within this area, we selected five independent forest landscapes (or circular sampling regions of 0.5‐km radius) based on remote sensing data and field survey in 2017 (Figure 1a). The five landscapes were located in five villages called Jiaosate, Akeqiapuba, Yonghong, Kezierhaying, and Mahulemuhanarele, respectively from east to west and varied gradually in their amount of forest cover from 30% to 65%. The average distance between the nearest landscapes was 6 km. In each forest landscape, we established six 30 × 30 m^2^ sampling sites, resulting in a total of 30 sampling sites (Figure 1b).

### 2.2 Sampling

In each sampling site, we mainly collected arthropods in forest understory vegetation and on poplar leaves. The sampling was conducted twice at the maximum growth period of the plant in 2018 in June and July. Since the abundance of arthropods can change with the state of weather, arthropods were collected during the windless and dry days between 10:00 and 15:00 only. Arthropods in forest understory vegetation were sampled by sweep netting (Haenke et al., 2014; Rösch et al., 2013). One person holds a sweep net to capture arthropods along the diagonal of the sampling site, a total of 50 times, and another person sorted and identified arthropods to species level and recorded abundance of arthropods. For the investigation of poplar leaves, we randomly selected five poplar trees in each sampling site. One person holds a high‐branch shears (artificially modified high‐branch shears can ensure that the cutting branches do not drop) to cut tree branches in four directions at the highest possible position, and another person recorded not only abundance of arthropods but also the total number of leaves and the number of damaged leaves in the 50 cm length range of branches to calculate the leaf‐eating rate of defoliators. For the arthropods cannot be accurately identified during the sampling period, we collected a complete voucher specimen, stored it in 70% alcohol solution, and recorded the acquisition time, location, and other useful information for further identification.

At the beginning of July in 2018, we quantified stand factors in each sampling site. Herbaceous species richness (number of herbaceous plant species) and the average height of herbaceous plants were recorded in five 1 × 1 m^2^ plots distributed along the diagonal of the sampling site. We also measured the mean diameter at breast height (DBH) and the average height of five selected poplar trees (up to a height of 15 m). These stand factors can provide good indicators of our site condition.

### 2.3 Landscape metrics and mapping

Landscape complexity was quantified within three radii of 100, 200, and 300 m around each sampling site based on high‐resolution remote sensing images (10 m) provided by the European “Sentinel‐2A” satellite (Figure 1b). First of all, we collected adequate ground truth data in June 2018 to rectify the results of image interpretation using Registration for ENVI 5.3 and kept sampling site at a minimum distance of 300 m to avoid spatial overlap. In addition, we preprocessed the remote sensing images to eliminate errors and improve their quality and classified the land cover types of these forest landscapes into four categories: woodland, farmland, grassland, and wasteland by combining the results of image interpretation and field survey (Figure 1b). Finally, ArcGIS 10.3 was used to convert the format of five classification maps of forest landscape.

We classified the following four landscape metrics into three components of landscape complexity (Macfadyen et al., 2015) and measured them using Fragstats 4.2. We measured compositional landscape complexity using the Simpson's diversity index (SIDI) calculated at the landscape level with all four land cover categories. As a measure of configurational landscape complexity, we used mean shape index (SHAPE_MN) because the complexity in the shape of habitat patches may affect the arthropod communities via edge effects. In order to quantify landscape connectivity, landscape division index (DIVISION) was calculated to represent the degree to which a patch type is broken up into separate patches, and the structural connectivity of forest landscape was quantified by calculating patch cohesion index (COHESION) at the landscape level. Because nonhost plants can influence arthropod communities in other studies (Jactel, Birgersson, Andersson, & Schlyter, 2011; Straub et al., 2013), the proportion of nonhost plants and two other important stand factors, the distance from sampling site to the nearest river and the nearest village, were also quantified by using Fragstats 4.2.

### 2.4 Data analysis

All statistical analyses were performed using R version 3.5.1 (R Development Core Team, 2018). The abundance of poplar arthropods per sampling site from two aspects and the two sample dates were summed for statistical analyses.

Effects of landscape complexity and stand factors on the abundance and diversity of poplar arthropods were analyzed using linear mixed‐effect models (LMMs) and model averaging. LMMs (function “lme” in the R package nlme, Pinheiro, Bates, DebRoy, Sarkar, & Team, 2014) were conducted for (a) the abundance of poplar arthropod species and different arthropod guilds; (b) species diversity of the defoliator guild and the predator guild, represented by the Shannon diversity index; and (c) the average leaf‐eating rate of defoliators per sampling site. The abundance of poplar arthropod species was log(*x* + 1)‐transformed to reduce the influence of very abundant species (Pinheiro et al., 2014; Schirmel et al., 2018). Because both of herbaceous species richness and the distance from sampling site to the nearest river displayed a strong correlation (Spearman's correlation coefficients>|0.7|) with some variables at three spatial scales, we excluded them from future analyses. Explanatory variables in all full models were four landscape metrics, the remaining stand factors, and the proportion of nonhost plants. All of them were assigned as fixed effects, and the sampling region (forest landscape) was assigned as a random effect to account for the nested design of the sampling as local vegetation management of each sampling region is not identical (Dominik et al., 2018; Inclán et al., 2015; Perović et al., 2015).

In order to exclude all nonsignificant effects or interactions from the full models, we used an information‐theoretic approach to multimodel inference (Grueber, Nakagawa, Laws, & Jamieson, 2011). First of all, we standardized the input explanatory variables using function “standardize” in the arm package(Gelman & Su, 2016), as this will be essential for interpreting the parameter estimates after model averaging. We then calculated variation inflation factors (VIF) to examine collinearity among the explanatory variables and found no significant correlation (VIF values < 2.0 indicating low collinearity) (Schirmel et al., 2018). Akaike's information criterion (AICc), corrected for small sample size, was used to determine the most effective spatial scale (the scale containing the model with the lowest AICc value across three spatial scales) for the prediction of arthropod communities and rank candidate models. The functions “dredge” and “get.models” implemented in the MuMIn package (Barton, 2015) were used to generate a total model set of all possible models and select the best‐fit model (with the minimum AICc value) and top‐ranked models (ΔAICc < 2) at each spatial scale. At the most effective scale, model averaging was performed using the function “model.avg” to produce averaged parameter estimates from the top model set (Gallé et al., 2018). The model‐averaged results showed the relative importance (quantified by the sum of the Akaike weights associated with each variable in the models in the top model set) of the explanatory variables (Grueber et al., 2011). The p‐values are based on the model‐averaged estimates and Ses (Schirmel et al., 2018), and “*p* < 0.001” was considered as very significant statistically.

## 3 RESULTS

Across all five forest landscapes, seven poplar arthropod species and 1,617 individuals were collected during the two sampling periods. Poplar defoliators represented 20.1% of all arthropods and were dominated by the green leafhoppers (*Cicadella viridis* L.), the poplar loopers (*Apocheima cinerarius* Erschoff), and the poplar leaf‐rolling weevils (*Byctiscus populi* L.). Predators accounted for 36.1% of the abundance of all arthropods and were mainly represented by dwarf spiders (Linyphiidae), lacewings of the Chrysopidae family (*Chrysopa phyllochroma* Wesmael), and carabid beetles from the genus *Calosoma* Weber, 1801. The polyphagous herbivores accounted for 43.8% of the abundance of all arthropods and were the migratory locusts (*Locusta migratoria* L.). In addition, the abundance of parasitoids was too small to analyze. The number of explanatory variables in all best‐fitting models was <7, and all best models included the combined effects of landscape variables and stand factors (Table 1). But these factors had different effects on different poplar arthropod communities (Table 2).

**Table 1:** Summary of the top‐ranked models (ΔAICc < 2) for estimating effects of the proportion of nonhost plants, the diversity of different habitat types (SIDI), shape complexity of habitat patches (SHAPE_MN), the connectivity of forest landscape (COHESION), the degree of landscape division (DIVISION), the average height of herbaceous plant (Herb height), the mean diameter at breast height (Mean DBH), the average height of poplar trees (Tree height), and the distance from sampling site to village (Dis. to village) on the abundance (log‐transformed) and diversity of poplar arthropod communities at the most effective spatial scale

	Scale	Top‐ranked models (ΔAICc < 2)	*df*	AICc	∆AICc	Weight
Abundance						
All defoliators	200 m	**Nonhost plants % + SIDI + COHESION+DIVISION + Herb height + Dis. to village**	**8**	**193.60**	**0.00**	**0.58**
		Nonhost plants % + SIDI + DIVISION + Herb height + Dis.to village	7	194.29	0.69	0.42
*Cicadella viridis*	200 m	**Nonhost plants % + SIDI + SHAPE_MN + COHESION + DIVISION + Mean DBH + Dis. to village**	**9**	**178.80**	**0.00**	**0.51**
		SIDI + SHAPE_MN + COHESION + DIVISION + Mean DBH + Dis. to village	8	180.03	1.23	0.28
		Nonhost plants % + SIDI + SHAPE_MN + COHESION + DIVISION + Dis. to village	8	180.60	1.81	0.21
*Apocheima cinerarius*	200 m	**Nonhost plants % + COHESION + DIVISION + Mean DBH + Tree height + Herb height**	**8**	**114.48**	**0.00**	**0.42**
		Nonhost plants % + COHESION + DIVISION + Mean DBH + Herb height	7	115.00	0.53	0.32
		Nonhost plants % + COHESION + DIVISION + Herb height	6	115.50	1.03	0.25
*Byctiscus populi*	200 m	**SIDI + Mean DBH + Herb height + Dis. to village**	**3**	**147.86**	**0.00**	**0.55**
		SIDI + Herb height + Distance to village	5	148.07	0.21	0.45
All predators	300 m	**Nonhost plants % + SHAPE_MN + COHESION + DIVISION + Dis. to village**	**7**	**332.55**	**0.00**	**0.64**
		Nonhost plants % + SHAPE_MN + COHESION + DIVISION + Dis. to village + Mean DBH	8	333.74	1.19	0.36
Linyphiidae	100 m	**SIDI + DIVISION + Dis. to village**	**5**	**141.09**	**0.00**	**0.33**
		SIDI + Dis. to village	4	141.45	0.36	0.28
		SIDI + COHESION + DIVISION + Dis. to village	6	141.88	0.79	0.22
		Nonhost plants % + SIDI + Dis. to village	5	142.48	1.39	0.17
*Chrysopa phyllochroma*	300 m	**SIDI + COHESION + DIVISION + Tree height + Herb height + Dis. to village**	**8**	**129.14**	**0.00**	**0.45**
		COHESION + DIVISION + Tree height + Herb height + Dis. to village	7	130.08	0.94	0.28
		COHESION + DIVISION + Herb height + Dis. to village	6	130.15	1.01	0.27
Carabidae	300 m	**Nonhost plants % + SIDI + SHAPE_MN + Mean DBH + Tree height + Dis. to village**	**8**	**288.65**	**0.00**	**0.40**
		Nonhost plants % + SIDI + SHAPE_MN + DIVISION + Mean DBH + Tree height + Dis. to village	9	289.04	0.39	0.33
		SIDI + SHAPE_MN + DIVISION + Mean DBH + Tree height + Dist. to village	8	289.42	0.78	0.27
*Locusta migratoria*	200 m	**SIDI + SHAPE_MN + DIVISION + Mean DBH + Herb height**	**7**	**297.12**	**0.00**	**0.53**
		SHAPE_MN + Mean DBH + Herb height	5	297.35	0.23	0.47
Diversity						
All defoliators	200 m	**Nonhost plants % + DIVISION + Mean DBH + Tree height + Herb height + Dis. to village**	**8**	**436.03**	**0.00**	**0.41**
		Nonhost plants % + COHESION + Mean DBH + Tree height + Herb height + Dis. to village	8	436.33	0.30	0.36
		Nonhost plants % + Mean DBH + Tree height + Herb height + Dis. to village	7	437.19	1.16	0.23
All predators	300 m	**Nonhost plants % + SIDI + COHESION + DIVISION + Mean DBH + Herb height + Dis. to village**	**9**	**328.67**	**0.00**	**0.63**
		Nonhost plants % + SIDI + COHESION + DIVISION + Mean DBH + Herb height + Dis. to village	8	329.76	1.09	0.37
Leaf‐eating rate	200 m	**Nonhost plants % + Mean DBH**	**4**	**189.45**	**0.00**	**0.40**
		Nonhost plants % + DIVISION + Mean DBH	5	190.58	1.13	0.23
		Nonhost plants % + COHESION + DIVISION + Mean DBH	6	190.78	1.33	0.21
		Mean DBH	3	191.3	1.85	0.16

Note

The best‐fit models for response variables are shown in bold; AICc, Akaike's information criterion corrected for small sample size; ΔAICc, difference in AICc value between the best ranked models; *df*, degree of freedom for each model; weight, Akaike weight, which can be interpreted as the conditional probabilities for each model.

**Table 2:** Results of model averaging to estimate effects of the proportion of nonhost plants, the diversity of different habitat types (SIDI), shape complexity of habitat patches (SHAPE_MN), the connectivity of forest landscape (COHESION), the degree of landscape division (DIVISION), the average height of herbaceous plant, the mean diameter at breast height, the average height of poplar trees, and the distance from sampling site to village on the abundance (log‐transformed) and diversity of poplar arthropod communities at the most effective spatial scale

	Scale (m)	The proportion of nonhost plants		The diversity of habitat types (SIDI)		Shape complexity of patches (SHAPE_MN)		Connectivity of forest landscape (COHISON)		Degree of landscape division (DIVISION)		The average height of herbaceous plant		The mean diameter at breast height		The average height of poplar trees		Distance from sampling site to village	
		Estimate	*p*	Estimate	*p*	Estimate	*p*	Estimate	*p*	Estimate	*p*	Estimate	*p*	Estimate	*p*	Estimate	*p*	Estimate	*p*
Abundance																			
All defoliators	200	−0.481	<2.00e−16	−1.821	<2.00e−16							0.560	2.18e−05						
*Cicadella viridis*	200			−2.793	1.00e−08	−0.855	0.0001											1.229	<2.00e−16
*Apocheima cinerarius*	200	−1.313	0.0001											2.881	0.0003				
*Byctiscus populi*	200			−0.719	0.0007														
All predators	300							0.565	0.0007	1.100	0.0006							0.556	1.00e−07
Linyphiidae	100																	0.660	0.0002
*Chrysopa phyllochroma*	300							4.360	5.50e−06	7.346	<2.00e−16								
Carabidae	300					1.782	2.60e−06			0.417	0.0002							1.500	0.0005
*Locusta migratoria*	200					−0.430	0.0002							0.562	<2.00e−16	−0.609	<2.00e−16		
Diversity																			
All defoliators	200	−0.389	<1.69e−05									0.639	<2.00e−16	0.445	<2.00e−16	0.613	<2.00e−16		
All predators	300							1.057	<2.00e−16	1.766	<2.00e−16							0.398	<2.00e−16
Leaf‐eating rate	200	−0.202	0.0001											0.431	4.00e−06				

Note

Effect sizes of the estimates have been standardized; “*p* < 0.001” is considered as very significant statistically.

### 3.1 Landscape complexity

**Figure 2:**
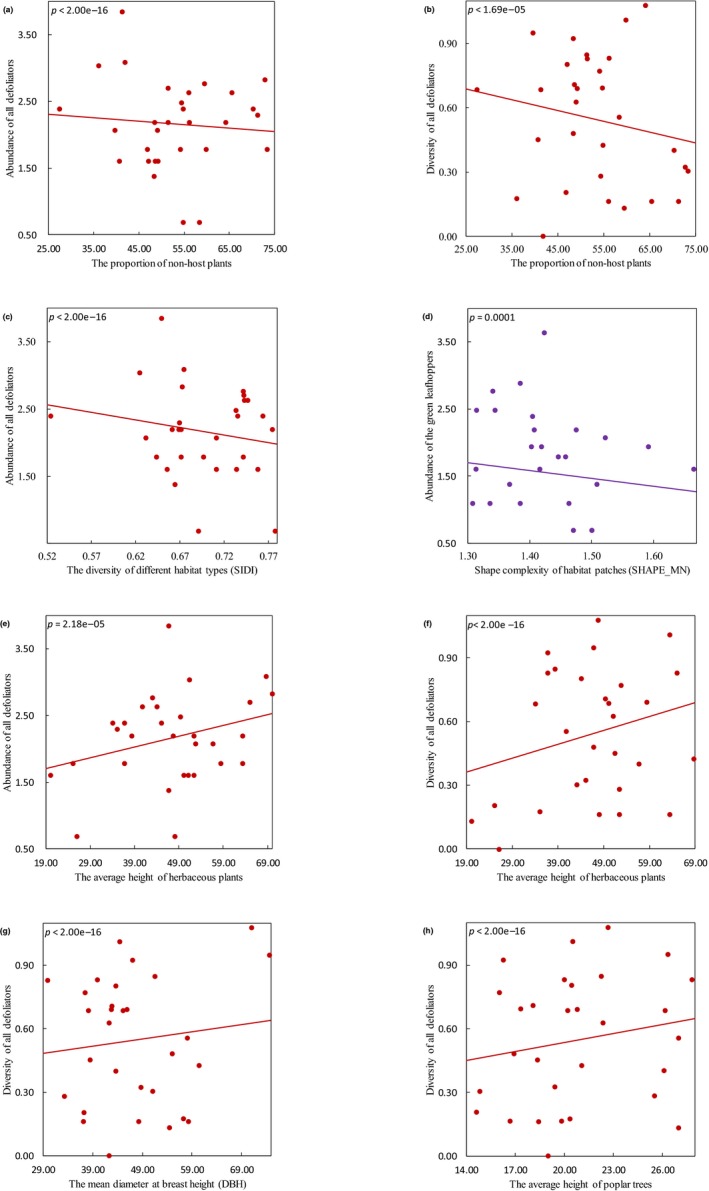
Very significant effects of the proportion of nonhost plants, landscape complexity, and stand factors on abundance (log‐transformed) and diversity of poplar defoliators. Interaction plots showing the relationships between (a) the proportion of nonhost plants and abundance of all defoliators, (b) the proportion of nonhost plants and diversity of all defoliators, (c) the diversity of different habitat types (SIDI) and abundance of all defoliators, (d) shape complexity of habitat patches (SHAPE_MN) and abundance of the green leafhoppers (*Cicadella viridis* L.), (e) the average height of herbaceous plants and abundance of all defoliators, (f) the average height of herbaceous plants and diversity of all defoliators, (g) the mean diameter at breast height (DBH) and diversity of all defoliators, (h) the average height of poplar trees and diversity of all defoliators

The proportion of nonhost plants negatively influenced the abundance (Estimate = −0.481, *p* < 2.00e−16) and diversity (*E* = −0.389, *p* < 1.69e−05) of all defoliators (Figure 2a,b), the abundance of the defoliator *Apocheima cinerarius* Erschoff (*E* = −1.313, *p* = 0.0001), and the average leaf‐eating rate (*E* = −0.202, *p* = 0.0001). The compositional landscape complexity (landscape diversity), represented by Simpson's diversity index (SIDI), was negatively correlated with the abundance of all defoliators (*E* = −1.821, *p* < 2.00e−16) (Figure 2c). The abundance of both *Cicadella viridis* L. (*E* = −2.793, *p* = 1.00e−08) and *Byctiscus populi* L. (*E* = −0.719, *p* = 0.0007) also declined with increasing landscape diversity. The spatial scale at which defoliators best responded to the proportion of nonhost plants and landscape diversity was defined at 200 m (Table 2). However, we found no significant effect of these two factors on predator communities.

**Figure 3:**
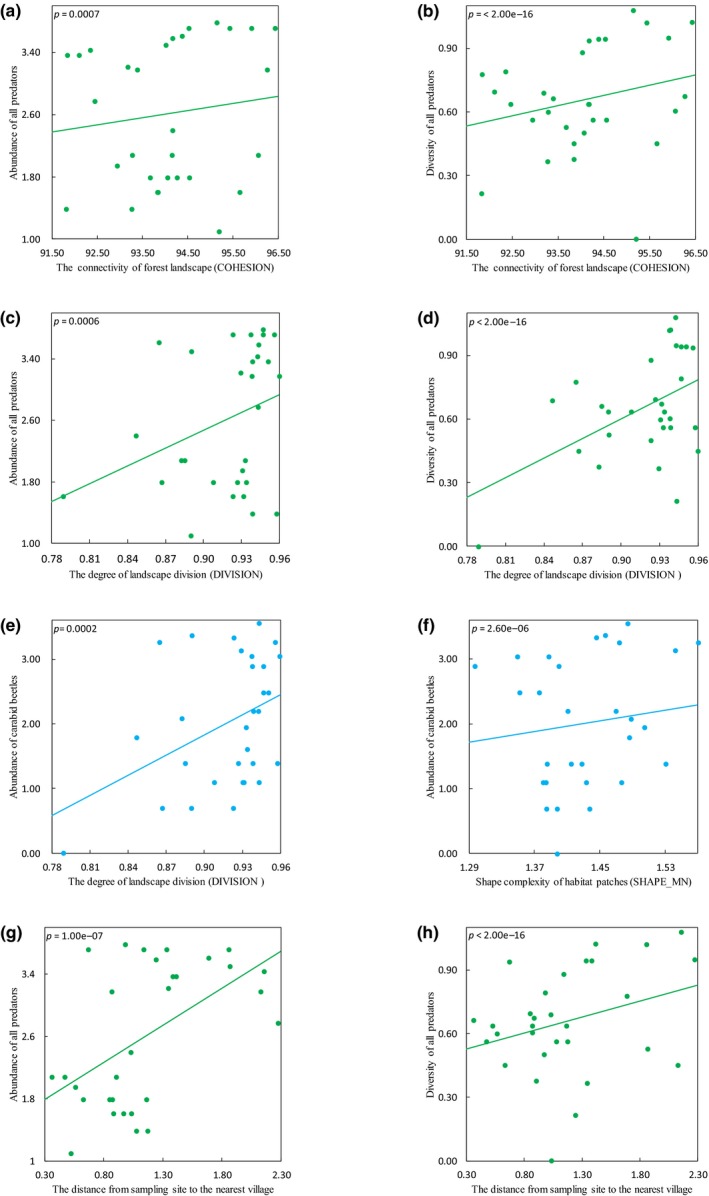
Very significant effects of the proportion of nonhost plants, landscape complexity, and stand factors on abundance (log‐transformed) and diversity of predators. Interaction plots showing the relationships between (a) the connectivity of forest landscape (COHESION) and abundance of all predators, (b) the connectivity of forest landscape (COHESION) and diversity of all predators, (c) the degree of landscape division (DIVISION) and abundance of all predators, (d) the degree of landscape division (DIVISION) and diversity of all predators, (e) the degree of landscape division (DIVISION) and abundance of carabid beetles from the genus *Calosoma* Weber, 1801, (f) shape complexity of habitat patches (SHAPE_MN) and abundance of carabid beetles from the genus *Calosoma* Weber, 1801, (g) the distance from sampling site to the nearest village and diversity of all predators, (h) the distance from sampling site to the nearest village and diversity of all predators

The configurational landscape complexity influenced differently poplar defoliators and predators. The abundance of two poplar defoliators,* Cicadella viridis* L. (*E* = −0.855, *p* = 0.0001) (Figure 2d) and *Locusta migratoria* L. (*E* = −0.430, *p* = 0.0002), declined with increasing shape complexity of habitat patches (SHAPE_MN). But only the abundance of carabid beetles from the genus *Calosoma* Weber, 1801, increased with increasing shape complexity of habitat patches (*E* = 1.782, *p* = 2.60e−06) (Figure 3f).

As a measure of landscape connectivity, both of patch cohesion index (COHESION) and landscape division index (DIVISION) calculated at the landscape level promoted predator communities at the scale of 300 m (Table 2). The structural connectivity of forest landscape (COHESION) increased the abundance (*E* = 0.565, *p* = 0.0007) and diversity (*E* = 1.057, *p* = <2.00e−16) of all predators (Figure 3a,b). The abundance of lacewings of the Chrysopidae family (*E* = 4.360, *p* = 5.50e−06) also increased with the structural connectivity of forest landscapes (Figure 3e). The degree of landscape division (DIVISION) positively influenced the abundance (*E* = 1.100, *p* = 0.0006) and diversity (*E* = 1.766, *p* < 2.00e−16) of all predators (Figure 3c,d). The abundance of both lacewings (*E* = 7.346, *p* < 2.00e−16) and carabid beetles (*E* = 0.417, *p* = 0.0002) also responded positively to the degree of landscape division.

The best spatial scale at which poplar arthropods responded to landscape complexity varied between arthropod species. Predators all responded to landscape complexity at the largest spatial scale (300 m) except dwarf spiders (Linyphiidae) (100 m), but all poplar defoliators were influenced by landscape complexity at a middle spatial scale (200 m) (Table 2).

### 3.2 Stand factors

We found significant correlations between stand factors and poplar arthropod communities. Both the abundance (*E* = 0.560, *p* = 2.18e−05) and diversity (*E* = 0.639, *p* < 2.00e−16) of all defoliators were positively correlated with the average height of herbaceous plants (Figure 2e,f). The mean diameter at breast height of poplar trees increased the abundance of poplar loopers of the genus *Apocheima* (*E* = 2.881, *p* = 0.0003), *Locusta migratoria* L. (*E* = 0.562, *p* < 2.00e−16), diversity of all defoliators (*E* = 0.445, *p* < 2.00e−16) (Figure 2g), and the average leaf‐eating rate (*E* = 0.431, *p* = 4.00e−06). The abundance of migratory locusts (*E* = −0.609, *p* < 2.00e−16) declined with increasing average height of poplar trees, but diversity of all defoliators (*E* = 0.613, *p* < 2.00e−16) was positively correlated with it (Figure 2h). Furthermore, the distance from sampling site to the nearest village positively influenced the abundance of *Cicadella viridis* L. (*E* = 1.229, *p* < 2.00e−16), all predators (*E* = 0.556, *p* = 1.00e−07) (Figure 3g), dwarf spiders (*E* = 0.660, *p* = 0.0002), carabid beetles (*E* = 1.500, *p* = 0.0005), and diversity of all predators (*E* = 0.398, *p* < 2.00e−16) (Figure 3h).

## 4 DISCUSSION

Landscape complexity and stand factors had different effects on different poplar arthropod communities. The proportion of nonhost plants and landscape diversity negatively influenced defoliator communities, but they had no effects on predators. In contrast, the abundance and diversity of predators increased with increasing landscape connectivity of forest landscapes, but defoliators had no significant response to it. Additionally, defoliators were positively correlated with the average height of herbaceous plants and size of host trees, and most predators were solely explained by the distance from sampling site to the nearest village.

### 4.1 Landscape complexity

Our results did not support our first hypothesis that the compositional landscape complexity increases the abundance and diversity of natural enemies; however, it negatively influenced the abundance of all defoliators and two defoliator species, *Cicadella viridis* Linnaeus and *Byctiscus populi* Linnaeus. This may be due to the fact that increasing diversity of surrounding habitat types reduces the proportion of woodland where poplar defoliators mainly inhabit, which is also consistent with our result that the proportion of nonhost plants reduces the abundance and diversity of all defoliators. The diversity of the surrounding vegetation has been reported to reduce herbivore damage to host plants in forest ecosystem (Jactel & Brockerhoff, 2007), and nonhost plant diversity was found to reduce potato leafhopper damage to alfalfa (Straub et al., 2013). Balzan et al. (2015) showed that the diversity of habitat type and field margin vegetation within the surrounding landscape reduced tomato damage caused by Lepidoptera pests. The absence of effects of habitat diversity on natural enemies may be explained by the fact that surrounding vegetation represents same important food and habitat resource as host trees for polyphagous predators.

Poplar arthropod communities strongly responded to the configurational complexity of forest landscape, especially the complexity in the shape of habitat patches. We found significant relationships between defoliators and shape complexity of habitat patches in our study, as patch shape can influence host finding for herbivores (Grez & Prado, 2000; Hamazaki, 1996). Patch shape is represented by the length of the perimeter of the patch. Patches with longer perimeters will encounter a wide variety of microhabitats and thus would negatively influence interior arthropod abundance (Muriel & Grez, 2002). Therefore, interior arthropods, such as green leafhoppers and migratory locusts, are likely to inhabit patches with more simple shapes because of the accessibility of food sources and refuge. But in agreement with our second hypothesis, natural enemies are more abundant in complex patch shapes, suggesting that the edge effect is important for carabid beetles in poplar forests in our study. Collinge and Palmer (2002) showed that movement of ground‐dwelling beetles across patch boundaries may be affected by patch shape. Habitat patch shape seems to have a significant effect on the community structure of predators by modifying their microhabitat choice (Cobbold & Supp, 2012). In a word, patch shape may play an important role among defoliators and natural enemies (Olson & Andow, 2008). And the higher abundance of natural enemies in the borders may affect defoliator inside patches (Grez & Prado, 2000).

Our results of significant positive effects of the connectivity of forest landscape on natural enemies did support our third hypothesis, which is in accordance with Coppolillo et al. (2004), who showed that the connectivity of a landscape was important for predators as they become increasingly dependent on movement between different habitat patches. Successful migration of predators, drived by connected habitat patches close enough, is a key aspect for their survival, and landscape connectivity need to be taken into account when studying their population dynamics and their response to landscape structure (Bodin & Norberg, 2007). So the abundance and diversity of all predators in our study increased with increasing the structural connectivity of forest landscape. We also found that the positive correlation between the degree of landscape division and most predators.

Predators are often less effective in searching for alternative food resources and shelter when the landscape consists of a single patch (landscape division index = 0) (Straub et al., 2013). Additionally, the degree of landscape division can describe the complexity of forest landscape and is the precondition of the structural connectivity of forest landscape. To conclude, the results of the positive correlation between the connectivity of forest landscape and natural enemies can lead to different approaches to manage a forest landscape (Baggio et al., 2011).

The most effective spatial scale at which poplar arthropods responded to the landscape complexity is different for each arthropod species. Such scale effect is primarily driven by their dispersal ability and thus vary across species (Jackson & Fahrig, 2012). Dispersal ability is seen as crucial for species conservation at the landscape level (Bertrand et al., 2016; Topping, 1999). Drapela et al. (2008) showed that differences in dispersal power of spider species lead to the scale dependency of relations. All poplar defoliators in our study are either wingless or limited in flight, so the scale at which they responded (200 m) is expected to be lower than for highly mobile natural enemies (300 m) as most predators live at the surrounding vegetation and can disperse to multiple habitat patches. But dwarf spiders responded to landscape complexity at a small scale (100 m) because they have low mobility. The landscape in the closer surroundings is supposed to be more important for species with low dispersal abilities (Schmidt, Roschewitz, Thies, & Tscharntke, 2005). An ability to predict scale effects of a landscape would make design of landscape study more efficient and would enable landscape managers to plan at the appropriate scale (Jackson & Fahrig, 2012).

### 4.2 Stand factors

In accordance with our hypothesis (4), we found that the abundance and diversity of different poplar arthropods responded differently to stand factors. The average height of herbaceous plants showed a positive effect on the abundance and diversity of all defoliators, which may be related to their oviposition site. In our study, most defoliators lay eggs on stems and leaves of the grass family (Poaceae). Zhang and Liu (2006) reported that experience of nonhost plants by phytophagous insects may alter their oviposition responses to these plants. The various processes surrounding insect oviposition are crucial to their population dynamics because the choice of oviposition site ultimately influences the survivorship and spatial distribution of their progeny (Lancaster, Downes, & Arnold, 2010). Arthropod communities living on poplar trees can be affected by the growth of their host plants in several ways. Tree height and stem DBH (diameter at breast height) are commonly used measures of tree growth (Sumida, Miyaura, & Torii, 2013). Campos, Vasconcelos, Ribeiro, Neves, and Soares (2006) suggested that there was a significant increase in both abundance and species richness of ants and insect herbivores with an increase in tree height. In our study, the average height of poplar trees enhanced diversity of all defoliator but reduced abundance of the migratory locusts, perhaps because the latter inhabited principally crops or grasslands and rarely fed on high leaves. Additionally, development of leaf area and leaf number of poplar partly depends on its DBH, and trees with high‐quality leaves tend to suffer from frequent herbivore attack. Gaku (2003) showed a clear relationship between leaf traits of willow and its susceptibility to herbivory. Thus, our results also indicate that diversity of all defoliators and the average leaf‐eating rate increase with increasing mean DBH of poplar trees. We also found that the distance from sampling site to the nearest village positively influenced arthropod communities, especially the predator guild. The abundance and diversity of all predators were associated with complex landscape structure. However, the intensification of grazing and landscape simplification reduces the diversity of habitat types. These processes are all potential contributors for the decline of predator abundance (Bertrand et al., 2016; Inclán et al., 2015). So a forest stand far from the village can enhance predators by maintaining the landscape complexity of habitats. Indeed, predators were largely driven by the distance to the nearest village, likely masking any effects of other stand factors.

### 4.3 Synthesis and applications

Species of the genus *Populus* can be found throughout the Northern Hemisphere and play an important role in the agroforestry systems (Bradshaw, Ceulemans, Davis, & Stettler, 2000). Poplar is also the main afforestation species in China. Various insect pests invading poplars not only affect the growth of trees but also sometimes threat their survival. There is no doubt that forest will eventually face many similar problems as agriculture in regard to pest control. However, a great variety of methods and tools that are widely used for pest control in agriculture are not available or are not suitable for application in forestry. Therefore, sustainable control of insect pests in natural forests will rely on the knowledge of biological control and on the integration of such knowledge across multiple scales from the local level to the landscape level. For the first time, we indicate that the multiscale effects of landscape complexity and stand factors on arthropod communities in poplar forest ecosystem. Our results provide evidence that the compositional complexity of surrounding landscape can reduce the abundance of defoliators and the shape complexity of habitat patches can enhance natural enemies. Predators with high dispersal ability such as lacewings can be affected by the connectivity of forest landscape at larger scales, while arthropods with low dispersal ability such as spiders benefit from some stand factors at a small scale. We therefore recommend that the management of forest landscapes for the conservation of natural enemies and the improvement of biological pest control should increase the diversity of surrounding habitat types and maintain more complex shape and structural connectivity of forest landscape within at least 100–300 m radii. With the growth of herdsman population, local vegetation management and grazing intensity can potentially affect the community structures of poplar arthropods, even leading to the reduction in the efficiency of biological pest control. The effects of vegetation management cannot be directly disentangled within the context of landscape complexity, but we indirectly accounted for these effects by a nested design in our statistical analysis. Furthermore, future research should quantify grazing intensity and assess its effect on poplar arthropod communities. We should also focus on the ecological traits of natural enemies in a future study as the higher functional diversity of predatory arthropods implies not only different hunting strategies, but also a higher potential for biological control of insect pests (Letourneau & Bothwell, 2008). Thus, the approach of functional diversity offers an available tool to assess crucial ecosystem service.

## AUTHORS' CONTRIBUTIONS

B.L.W. and C.M.T conceived the ideas and designed methodology; B.L.W. and J.L.S. collected the data; B.L.W. analyzed the data; B.L.W. led the writing of the manuscript. All authors contributed critically to the drafts and gave final approval for publication.

## Data Availability

Data available via the Dryad Digital Repository: https://doi.org/10.5061/dryad.7p0sf3n.
